# Idiopathic follicular mucinosis in childhood^☆^^☆☆^

**DOI:** 10.1016/j.abd.2019.06.010

**Published:** 2020-02-18

**Authors:** Fernanda José Bauer, José Roberto Paes de Almeida, Angelo Sementilli, Sandra Lopes Mattos e Dinato

**Affiliations:** Department of Dermatology, Centro Universitário Lusíada, Santos, SP, Brazil

Dear Editor,

Follicular mucinosis is a rare condition, belonging to the group of cutaneous mucinoses, characterized by localized or diffuse mucin deposits in the skin or within hair follicles. Two forms were described: primary (or idiopathic) and secondary form, which may be associated with benign or malignant conditions.^1^

The presence of well-defined papular, erythematous or reddish-brown erythema papules or plaques clinically characterize the condition. Follicular keratosis or alopecic patches could also be observed. Other less common forms have been described, such as acneiform, eczematous, cystic or nodular.^2^

The patient is an 11-year-old white male, with no relevant personal or family history, with a 2 year history of asymptomatic cutaneous lesion on the face.

On the dermatological examination: hypochromic lesion topped by follicular and nonfollicular shiny papules on the nasal, malar, zygomatic and left periorbital region and papular lesions on the upper right eyelid and mild left eyelid edema (Fig. 1). Sensory evaluation results are normal.

**Figure 1:**
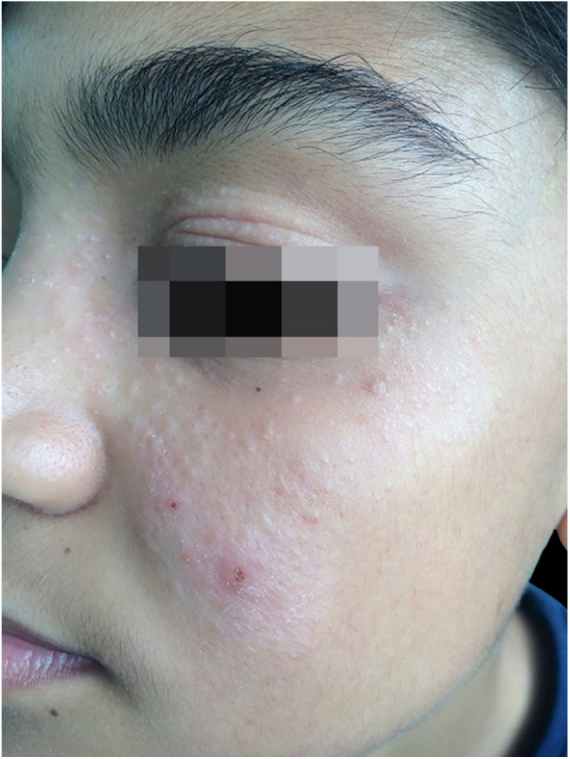
Hypochromic lesion topped by shiny papules.

A routine anatomopathological examination (Hematoxylin & eosin staining) reveals preserved epidermis. Dermis presents some hair follicles containing fibromyxoid stroma and mixed pattern inflammatory cells (Fig. 2). Alcian Blue stain reveals built-up mucin in the outer root sheath of the hair follicle (Fig. 3).

**Figure 2:**
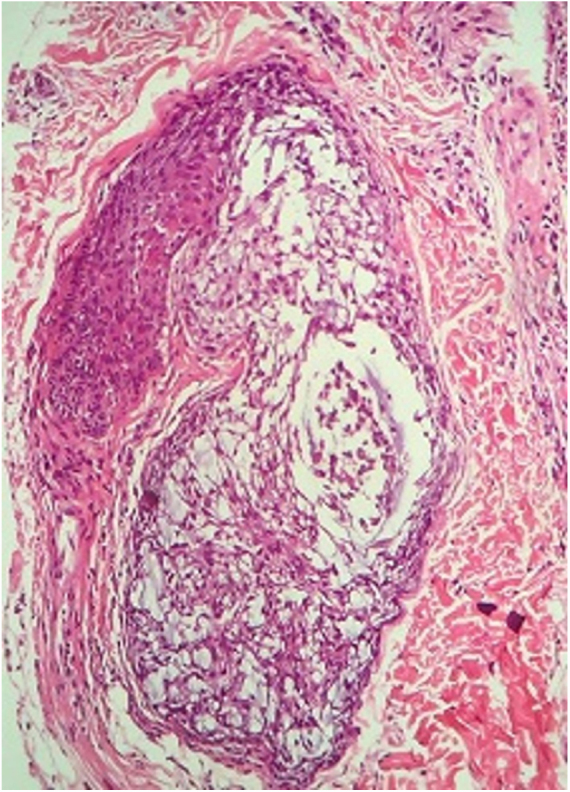
Hair follicles containing fibromyxoid stroma (Hematoxylin & eosin, ×100).

**Figure 3:**
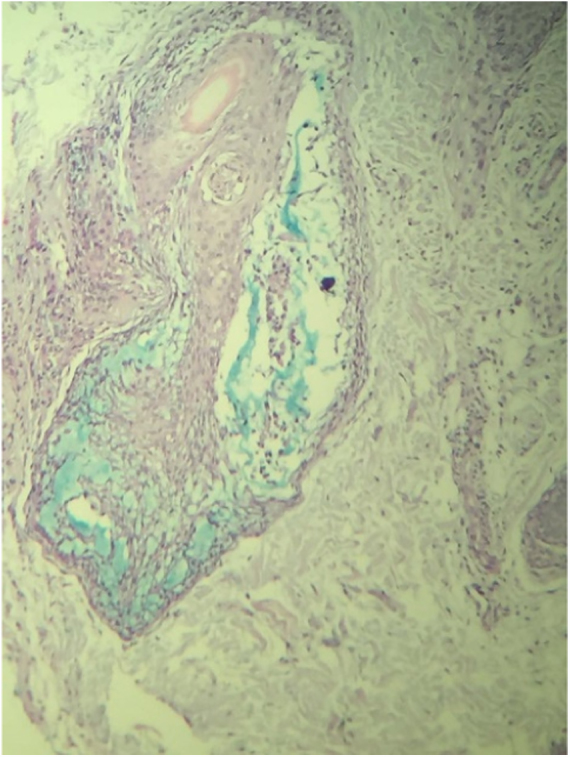
Mucin in the outer root sheath of the hair follicle (Alcian Blue, ×100).

Laboratory tests were requested to investigate associated diseases; all results were normal.

Treatment with daily application of high-potency topical corticosteroid showed improvement; however, recurrence occurred when discontinued.

In 1957, Hermann Pinkus described a group of 6 patients with localized alopecia, histopathologically characterized by mucin deposits in the hair follicles, which he named alopecia mucinosa. Jablonska et al. proposed in 1959 to change the name to follicular mucinosis, a term accepted to this day.^3^

Its cause is still unknown. Today, it is considered a standard reaction of the follicular epithelium to various factors.^1^

This dermatosis can have two clinical forms:^1^, ^2^, ^4^

1. Primary form: an idiopathic, benign and transient form, which commonly occurs in children and adults. In younger patients, it usually affects the head and neck, receding spontaneously after 2–24 months in most cases. Some rare cases of developing Hodgkin's disease, other lymphomas and leukemia have been recorded. Adult patients present generalized lesions that may last indefinitely.^4^
2. Secondary form: normally affects adults and older patients, and is associated with an underlying inflammatory or neoplastic condition. The most common is mycosis fungoides.^4^

In the reported case, clinical and lab analysis allowed to exclude associated diseases and diagnose the condition as the idiopathic form of follicular mucinosis.

Histopathology is essential for diagnosis since it shows mucin deposits on the outer root sheath of the hair follicle, in addition to inflammatory infiltrates composed of lymphocytes, macrophages and eosinophils with folliculotropic lymphocytes. The benign form is determined by the extension of the eosinophilic inflammatory infiltrate and significant mucinous alterations in the follicular epithelium.^5^ Similar findings were observed in the patient‘s results. Presence of epidermotropic lymphocytes and dense perifollicular infiltrate of atypical cells suggest a malignant form of the condition; however, no evidence of it was found in the studied patient.

It's interesting to mention that the chances of lymphomas appearing in follicular mucinosis patients varies greatly. The criteria adopted in the different evaluations change ranging from 14% to 32%. For example, Coskey and Mehregan detected lymphoma in 7 out of 50 follicular mucinosis patients (14%), Emmerson in 8 out of 47 patients (17%), Logan and Headington in 21 out of 80 patients (26%), Mehregan et al. in 9 out of 33 patients (27.2%) and Gibson et al. in 19 out of 59 patients (32%).^1^

On the other hand, the frequency of the disease among patients with cutaneous lymphomas has not been established in literature. Marti et al. found the disease in 5 out of 43 patients (11%).^1^

Regarding treatment, there is no specific medication for idiopathic follicular mucinosis. However, topical, intralesional and systemic corticosteroids, in addition to dapsone, antimalarial, isotretinoin and minocycline, can be used.^4^ Treatment was done using topical corticosteroids; the lesions improved but had a slight recurrence. These patients require periodic monitoring.

The importance of the report is that is shows a rare incidence of follicular mucinosis in childhood. It is imperative to emphasize the obligation of long-term monitoring in order to completely rule out the connection with associated neoplasias.

## Financial support

None declared.

## Authors’ contributions

Fernanda José Bauer: Conception and planning of the study; elaboration and writing of the manuscript; obtaining, analysis, and interpretation of the data; critical review of the literature; critical review of the manuscript.

José Roberto Paes de Almeida: Approval of the final version of the manuscript; conception and planning of the study; effective participation in research orientation; intellectual participation in the propaedeutic and/or therapeutic conduct of the studied cases; critical review of the literature; critical review of the manuscript.

Angelo Sementilli: Obtaining, analysis, and interpretation of the data.

Sandra Lopes Mattos e Dinato: Approval of the final version of the manuscript.

## Conflicts of interest

None declared.
